# Relationship between Serum 25(OH)D and Depression: Causal Evidence from a Bi-Directional Mendelian Randomization Study

**DOI:** 10.3390/nu13010109

**Published:** 2020-12-30

**Authors:** Anwar Mulugeta, Amanda Lumsden, Elina Hyppönen

**Affiliations:** 1 Australian Centre for Precision Health, University of South Australia Cancer Research Institute, University of South Australia, Adelaide, SA 5000, Australia; anwarmulugeta.gebremichael@unisa.edu.au (A.M.); amanda.lumsden@unisa.edu.au (A.L.); 2 Department of Pharmacology and Clinical Pharmacy, College of Health Sciences, Addis Ababa University, Addis Ababa 1000, Ethiopia; 3 South Australian Health and Medical Research Institute, Adelaide, SA 5000, Australia; 4 Unit of Clinical and Health Sciences, University of South Australia, Adelaide, SA 5000, Australia; 5 Population, Policy and Practice, UCL Great Ormond Street Institute of Child Health, London WC1N 1EH, UK

**Keywords:** 25(OH)D, nutritional vitamin D status, depression, observational analysis, Mendelian randomization, UK Biobank

## Abstract

The relationship between depression and vitamin D deficiency is complex, with evidence mostly from studies affected by confounding and reverse causality. We examined the causality and direction of the relationship between 25-hydroxyvitamin D (25(OH)D) and depression in bi-directional Mendelian randomization (MR) analyses using information from up to 307,618 white British participants from the UK Biobank and summary results from the SUNLIGHT (*n* = 79,366) and Psychiatric Genomics consortia (PGC 113,154 cases and 218,523 controls). In observational analysis, the odds of depression decreased with higher 25(OH)D concentrations (adjusted odds ratio (OR) per 50% increase 0.95, 95%CI 0.94–0.96). In MR inverse variance weighted (IVW) using the UK Biobank, there was no association between genetically determined serum 25(OH)D and depression (OR per 50% higher 0.97, 95%CI 0.90–1.05) with consistent null association across all MR approaches and in data from PGC consortium. In contrast, genetic liability to depression was associated with lower 25(OH)D concentrations (MR IVW −3.26%, −4.94%–−1.55%), with the estimates remaining generally consistent after meta-analysing with the consortia. In conclusion, we found genetic evidence for a causal effect of depression on lower 25(OH)D concentrations, however we could not confirm a beneficial effect of nutritional vitamin D status on depression risk.

## 1. Introduction

Studies suggest a link between hypovitaminosis D and a diverse range of conditions, including an increased risk of depression [1,2]. Vitamin D receptors (VDR) and the enzymes required for local vitamin D activation are expressed in the brain [3] and there are plausible biological mechanisms that could mediate an effect of vitamin D deficiency to depression. For example, hormonal vitamin D promotes the production of neurotransmitters including serotonin, dopamine and norepinephrine, through VDR-mediated transcriptional upregulation of hydroxylase genes that catalyse their synthesis [4,5]. Decreased levels of these neurotransmitters are associated with depression, and drugs that promote their availability represent effective antidepressant treatments. Active vitamin D also has anti-inflammatory properties, which may counter the increase in inflammatory cytokines associated with depression [1]. However, results from randomized control trials (RCT) investigating potential benefits of vitamin D supplementation in depression have been inconsistent. A recent large-scale RCT [6] and an earlier systematic review and meta-analysis including nine RCTs (*n* = 4923), investigated the effects of vitamin D supplementation in reducing depressive symptoms in adults, but found no effects [7]. In contrast, another review comparing 15 RCTs, and assessing the studies based on their methodological merit, found that vitamin D supplementation produced favourable results in six of seven studies ‘without biological flaws’, but only in three of the eight studies ‘with flaws’; in which the ‘flaws’ were defined as lack of 25(OH)D measures or reduced 25(OH) levels in the intervention group, and those with baseline 25(OH) level indicated sufficiency [8]. Beneficial effects of vitamin D supplementation on depression have been observed when used as adjunct treatment with antidepressants [9], and amongst patients with psychiatric disorders [10]. While the benefits of vitamin D in the prevention of depression remain uncertain, it is also possible that the observational association between 25(OH)D and depression can be due to links between behaviour and vitamin D-related covariates. Indeed, many of the characteristics and behaviours that associate with depression, such as obesity, lower use of vitamin supplements, poor-quality diet, and less time spent outdoors, can also limit vitamin D intakes from the diet and/or sunlight-induced synthesis in the skin [11].

**Figure 1:**
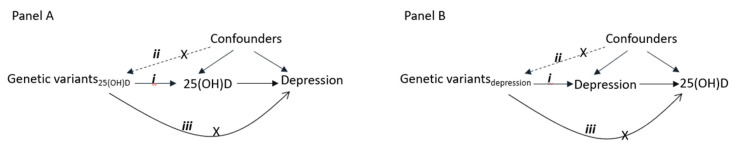
Diagram showing the principle of Mendelian randomization (MR) analysis [15]. Panel A shows the association between 25(OH) and depression, and Panel B between depression and 25(OH)D. The numbers reflect the MR assumptions that the genetic variants should (i) be associated with the exposure, (ii) not associated with the confounders of exposure-outcome association, and (iii) not affect the outcome through pathways other than exposure.

Currently, it is uncertain whether the relationship between 25(OH)D concentrations and depression is causal, and whether it operates in one or both directions. Mendelian randomization (MR) is a genetic approach that allows for the testing of causality even in situations where clinical trials may be difficult to conduct. As it approximates the exposure using genetic variants that are determined at the time of conception (which do not change in response to health or lifestyles), it allows us to overcome common problems of observational studies including reverse causality and confounding (Figure 1). Prior MR studies have been either unidirectional (i.e., 25(OH)D to depression) or based on information only from GWAS summary data [12,13,14]. In this study, we use information from up to 307,618 participants from the UK biobank together with summary level data from consortia meta-analyses to investigate the causality and direction of the association between 25(OH)D and depression. If lower 25(OH)D has a causal effect on depression, genetically instrumented lower 25(OH)D concentration should be associated with a proportionately greater risk of depression. In the other direction, if depression leads to low vitamin D status, genetic variants associated with depression risk should associate with lower 25(OH)D concentrations.

## 2. Methods

### 2.1. Participants

UK Biobank is a large prospective cohort of over 500,000 individuals aged 37–73 years during recruitment in one of the 22 assessment centres between 2006 and 2010 [16]. The cohort contains an extensive range of genetic and phenotypic data including information on lifestyle factors, diseases, various physical measures, and blood and urine biomarkers. A detailed description of the cohort can be found elsewhere [16,17]. The analysis for this study was restricted to unrelated white British ancestry participants (as evidenced by self-report and genetic analysis [17]) for whom complete genetic information is available (*n* = 337,484; Figure S1). For MR analysis investigating effects of 25(OH)D on depression, we included 251,962 participants with complete information on depression. We excluded participants (*n* = 85,522) for whom depression status was unclear, including participants who reported less than two weeks duration of depression/unenthusiasm (*n* = 429) and those who lacked this information (*n* = 85,093) [18]. Participants with complete information on 25(OH)D (*n* = 307,618) were used for the MR analysis of the effect of depression on 25(OH)D. For observational analyses, data were used from 229,832 participants for whom both 25(OH)D and depression information was available. We also used summary data from the SUNLIGHT consortium (*n* = 79,366) [19] and Psychiatric Genetic Consortium (113,154 cases and 218,523 controls), which together with the data from UK Biobank allowed us to conduct MR analyses for the effect of 25(OH)D on depression in up to 424,967 participants, and analyses of depression on 25(OH)D in up to 386,984 participants.

UK Biobank obtained informed consent from each participant, and ethical approval was granted by the National Information Governance Board for Health and Social Care and North West Multicentre Research Ethics Committee [20]. The current study is approved by the UK Biobank under application number 10171.

Depression was defined using information from touchscreen questionnaires, nurse-led interviews, and linked hospital registry records. Participants who had seen a general practitioner or a psychiatrist for anxiety, tension, nervousness or depression, and reported depression or unenthusiasm of at least two weeks duration were recoded as having depression [18]. Additional cases were identified from hospital diagnoses (ICD-10 F32 or F33 or the corresponding ICD-9 codes) obtained from Hospital Episode Statistics (HES). Individuals in the control group were those who had not seen a general practitioner or psychiatrist for anxiety, tension, nervousness or depression, and who had no hospital diagnosed depression, and no self-reported depression.

25(OH)D concentrations were measured from blood samples taken at baseline assessment with details on sample storage, processing, analysis and related quality controls reported elsewhere [21,22]. As covariates in the observational analyses, we included information on age, sex, assessment centre, date of blood sample collection, socioeconomic and lifestyle variables to account for potential confounding. Covariates were based on self-reported data from the baseline assessment, with the exception of the Townsend index reflecting area deprivation, which was derived from participants’ post codes as recorded in the National Health System primary care trust registries [23]. Education was based on highest qualification and grouped as “none”, “A-levels and below” and “degree or professional”. Based on the employment status and working hours, we grouped employment in to six categories as “lowest (first quartile) working hour group”, “second quartile”, “third quartile” and “highest (fourth quartile) working hour”, “retired” and “not working” groups. Body mass index (BMI) was calculated as weight (kg)/height^2^ (m^2^) and categorized based on World Health Organization recommendation [24] as “underweight” (<18.5 kg/m^2^), “normal” (≥18.5 kg/m^2^ and <25 kg/m^2^), “overweight” (≥25 kg/m^2^ and <30 kg/m^2^) and “obese” (≥30 kg/m^2^). Physical activity was grouped as “none”, “light/moderate”, and “strenuous activity”; smoking and alcohol consumption as “never”, “previous”, and “current”; and long-standing illness, disability or infirmity (hereafter referred as “long standing illness”) as “No” and “Yes”. Participants were asked about the frequency of oily or non-oily fish, and cheese consumption, that were categorized here as “never”, “less than once a week”, “once a week”, and “more than once a week”. Participants were asked about dietary restrictions, which were grouped here as “no egg or dairy containing food”, “no wheat containing food”, “no sugar, or sugar containing food”, and “eat all above”. Information regarding time spent outdoors in a typical day of summer and winter (“none”, “less than an hour”, “one”, “two”, “three”, “four”, “five”, “at least six hours”), and sun protection use (“never/rarely”, “sometimes”, “most of the time”, “always”, and “do not go out in sun”), were included in the covariates.

### 2.2. Genetic Variants of Serum 25(OH)D and Depression

Genotyping, imputation and related quality control were performed by the UK Biobank genetic team, for which detailed information can be found elsewhere [17]. Genotyping was done using UK BiLEVE (*n* ~ 50,000) and UK Biobank axiom array (*n* ~ 450,000), with the two arrays having 95% similarity in marker contents. Imputation was performed using the Haplotype reference consortium, and UK10K and 1000 genome reference panels. We used the third release UK Biobank genetic imputation dataset for extracting the genetic variants of interest.

We used six variants associated with serum level of 25(OH)D that attained genome-wide significance in a recent genome wide association study (GWAS), with these variants explaining ~3% of the variability in serum level of 25(OH)D [20]. In UK Biobank participants, these variants were in Hardy-Weinberg equilibrium (*p* > 0.17) and had minor allele frequency (MAF) of at least 0.18, and good imputation quality (INFO score ≥ 0.96) (Table S1). In a sensitivity analysis, we used 122 variants (excluding insertion and deletion type of genetic variants) associated with 25(OH)D that are identified in a recent GWAS that included UK Biobank [25].

We used 44 variants from a recent meta-analysis of GWAS on major depressive disorder (MDD) to instrument depression [26]. As the UK Biobank contributed to 10% of the cases and 4% of the controls in this GWAS meta-analyses, to minimize potential bias from sample overlap [27], we conducted sensitivity analyses using 17 MDD-related variants identified in an earlier MDD GWAS, that did not include UK Biobank participants [28]. All MDD-related variants were in Hardy-Weinberg equilibrium (*p* > 0.0003), with minor allele frequency greater than 0.07, and INFO score greater than 0.95 in the UK Biobank (Table S2 and Figure S2).

### 2.3. Statistical Analyses

We included observational and MR analysis approaches (Supplementary note and Figure S3) to investigate the bi-directional association between 25(OH)D and depression. Natural log-transformed 25(OH)D concentrations were used for analyses involving 25(OH)D as a continuous variable. In the model with 25(OH)D as the predictor, a converting factor of (1.50)^logOR^ was used to reflect the OR of depression per 50% change in 25(OH)D. In the model including 25(OH)D as the outcome, a conversion factor of 100 × (exp(beta)−1) enabled the effect estimate to be interpreted as percentage change (increase or decrease) in 25(OH)D by depression. Analyses were carried out using Stata v. 16.0 (StataCorp LLC, College Station, TX, USA) and R v. 3.5.0 (R Foundation for Statistical Computing, Vienna, Austria).

In the observational analysis, we tested whether the association between 25(OH)D and depression was different among males and females, and in different age groups, using related interaction terms. Where interactions were found, results are presented by sex and age stratification. We explored the association between serum 25(OH)D and depression using logistic regression with adjustment made for ranges of covariates in three models. The first model adjusted for basic covariates (age, sex, assessment centre, and blood sample collection date). The second model adjusted for basic plus socioeconomic-related covariates (Townsend deprivation, education, and employment). A final model additionally adjusted for lifestyle factors including smoking, alcohol consumption, BMI, physical activity, sun exposure in summer and winter, use of sun protection, diet restriction, fish and cheese consumption and long-standing illness. Using the same model structures, we investigated the association between depression and 25(OH)D. We conducted sensitivity analyses excluding serum 25(OH)D levels from aliquots affected by sample dilution bias (*n* = 7041) [22]. 25(OH)D levels were categorized into four groups defined as: <25 nmol/L, ≥25 and <50, ≥50 and <75 and >75 nmol/L for analysis involving 25(OH)D as a categorical indicator.

To provide genetic causal evidence of the 25(OH)D-depression bi-directional association, we used two-sample MR approaches using variant-exposure estimates from primary GWAS on 25(OH)D [20] and depression [26]. Variant-outcome estimates were derived both from the UK Biobank and from consortia meta-analyses [26], with estimates meta-analysed where relevant. The primary analyses used inverse variance weighted MR (MR IVW). As this approach assumes that there is no horizontal pleiotropy, i.e., that all the SNPs included are valid instruments, we conducted sensitivity analyses using four additional MR approaches, that allow one or more genetic variants to have horizontal pleiotropic effects, but which are relatively more power demanding. These include weighted median [29], weighted mode [30], MR-Egger [31], and MR-PRESSO [32], with each relying on different assumptions. The intercept from MR-Egger indicates the extent of directional pleiotropy [31]. Additional tests for pleiotropy and its impact on MR estimates were further explored using MR-PRESSO. MR-PRESSO involves three tests: MR-PRESSO global test, which detects the presence of unbalanced net-horizontal pleiotropy; MR-PRESSO outlier test, which identifies specific horizontal pleiotropic outlying variants; and MR-PRESSO distortion test, which assesses the change in the causal estimate after removal of pleiotropic outlying variants [32]. We also included leave-one-out analysis to examine whether the associations examined were sensitive to the effects by individual variants. In a sensitivity analyses involving 122 variants associated with 25(OH)D from recent GWAS [25], we collected the variant-25(OH)D association estimates from UK Biobank participants with no depression (from control) and the variant-depression association estimates from all UK biobank individuals to minimize the bias in the MR estimates from the sample overlap [27].

## 3. Results

Of the 229,832 individuals included in the observational analysis, 49.2% were females (Table 1). Generally, across the covariates, categories associated with lower 25(OH)D concentrations were also reflective of a higher prevalence of depression (Table 1 and Table S3). Individuals who were obese, not physically active, never consumed oily fish, had long-standing illness, or those who do not go in sunshine had lower 25(OH)D concentrations and higher prevalence of depression compared to the others.

The odds of depression were lower in individuals with higher compared to lower 25(OH)D and the association remained after adjusting for socioeconomic and lifestyle covariates (Table 2). Despite some evidence of interaction between 25(OH)D and sex on their association with depression (P_sex-interaction_ = 0.004), we saw evidence for a protective association both among males and females, with a slightly stronger association in males (Table S4). We did not find evidence for an interaction between 25(OH)D and age on depression (P_age-interaction_ = 0.07). Sensitivity analyses excluding serum 25(OH)D data affected by sample dilution bias provided consistent results with the analyses including all aliquots (Table S5).

**Figure 2:**
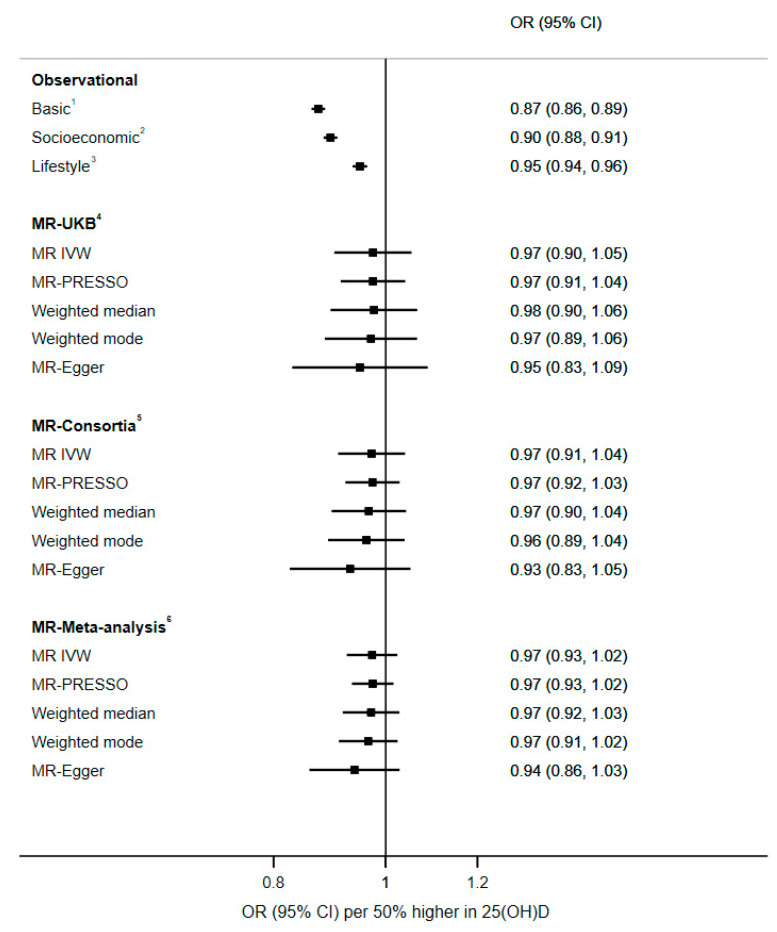
Observational and MR analyses on the association between 25(OH)D and the odds of depression. ^1^ Basic model included adjustment for basic covariates including age, sex, assessment centre and date of blood sample collected. ^2^ Socioeconomic model included adjustment for basic and socioeconomic-related covariates including education, Townsend deprivation index and employment. ^3^ Lifestyle model included adjustment for basic, socioeconomic and lifestyle-related covariates including smoking, alcohol consumption, BMI, physical activity, fish and cheese consumptions, dietary restriction, sun exposure [in summer or winter], use of sun protection, and long-standing illness. ^4^ MR analysis based on variant-depression association estimates from UK Biobank. ^5^ MR-analysis based on variant-depression association estimates from Wray et al. GWAS. ^6^ Meta-analysis of MR estimates from UK Biobank and Wray et al. GWAS. For all MR analysis, variant-25(OH)D estimates were from Jiang et al. GWAS. MR-Egger P-intercept (for all), *p* < 0.67.

We used six genetic variants from recent 25(OH)D GWAS in which the genetic risk score explained 2.7% of the variability in 25(OH)D in the UK Biobank (Figure S4). The results from two-sample MR analyses did not suggest a causal effect of 25(OH)D on depression (Figure 2). From MR IVW, the odds of depression per 50% higher genetically determined serum 25(OH) D were 0.97, with similar estimates from analyses using consortia, and estimates after meta-analyses; in all cases the confidence intervals crossed the null but also included the point estimate from observational analyses. The estimates from MR-PRESSO, weighted median, weighted mode and MR-Egger were consistent with the MR IVW estimate and CIs across all methods crossed the null (Figure 2). No evidence of directional pleiotropy was identified from MR-Egger intercept, MR-PRESSO outlier and leave-one-out analyses (Figure S5). We carried out further sensitivity analyses separating vitamin D according to those affecting 25(OH)D synthesis (*DHCR7*, and *CYP2R1*) and others, and again found no evidence for a causal association (Table S6). Extended analyses using 122 25(OH)D-related variants from recent GWAS [25] found no evidence of causal association between 25(OH)D and depression (Figure S6). Our study was powered (80%, alpha 0.05) to identify up to 8% lower odds of depression per 50% higher 25(OH)D.

**Figure 3:**
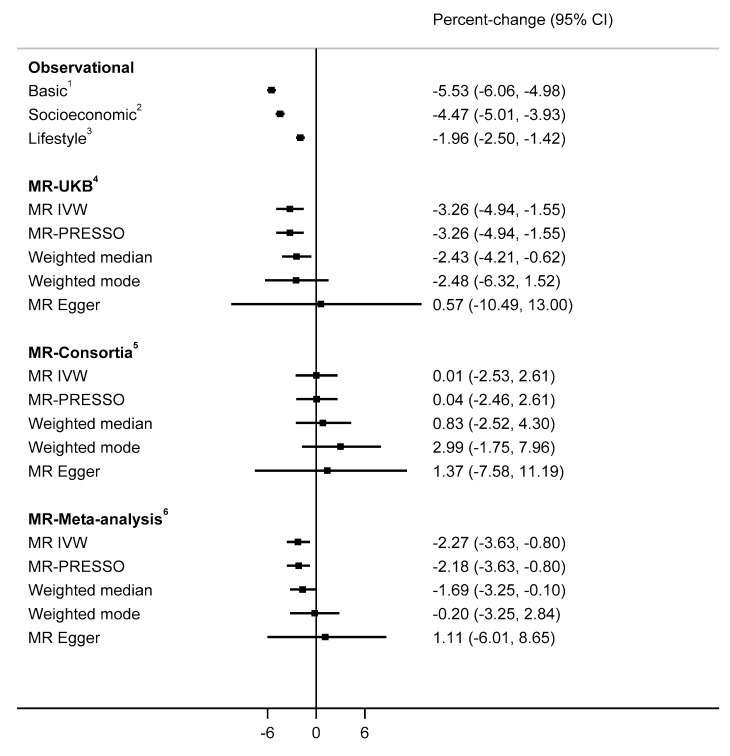
Observational and MR analyses on the association between depression and 25(OH)D concentrations. ^1^ Basic model included adjustment for basic covariates including age, sex, assessment centre and date of blood sample collected. ^2^ Socioeconomic model included adjustment for basic and socioeconomic-related covariates including education, Townsend deprivation index and employment. ^3^ Lifestyle model included adjustment for basic, socioeconomic and lifestyle-related covariates including smoking, alcohol consumption, BMI, physical activity, fish and cheese consumptions, dietary restriction, sun exposure [in summer or winter], use of sun protection and long-standing illness. ^4^ MR analysis based on variant-25(OH) association estimates from UK Biobank. ^5^ MR-analysis based on variant-25(OH)D association estimates from Jiang et al. GWAS. ^6^ Meta-analysis of MR estimates from UK Biobank and Jiang et al. GWAS. For all MR analysis, variant-depression estimates were from Wray et al. GWAS. MR-Egger P-intercept (for all), *p* < 0.51.

The observational analyses indicated that depression was associated with lower 25(OH)D concentrations (−1.96%, 95%CI −2.50 to −1.42; Figure 3). In MR IVW using 44 MDD-related variants from Wray et al. GWAS (Figure S4), serum 25(OH)D concentrations were 3.26% lower among individuals with genetically instrumented depression compared to others (Figure 3). Consistent evidence was found using MR-PRESSO and weighted median methods, while the effect estimate from weighted mode analysis were similar, but confidence intervals crossed the null. Despite the MR-Egger estimate being directionally inconsistent with estimates from other methods, the confidence interval was wide. We saw no evidence for horizontal pleiotropy in MR-Egger, MR-PRESSO outlier and leave-one-out analysis (Figure S7). Sensitivity analyses using 17 MDD-related variants that did not include UK Biobank participants (0.1% of the variability in MDD vs. 0.2% with 44 variants) gave causal estimates that were generally consistent with the primary analyses (Figures S8 and S9).

## 4. Discussion

Vitamin D is considered to be an important, modifiable risk factor for several diseases, and low vitamin D status is associated with depression. Despite consistent evidence from observational studies, the direction of the association and whether it is causal has been uncertain. Our study provided no evidence to support a causal role of vitamin D status on the risk of depression. In contrast, we found that depression leads to lower 25(OH)D concentrations, which may have clinical implications.

Our findings are consistent with those from a recent clinical trial that randomised 18,353 adults to receive vitamin D supplementation or placebo, which reported no differences in the risk of depression during the five years of follow-up [5]. In line with our study, earlier smaller MR studies have also not supported a causal effect of 25(OH)D on depression [12,13,14]. The effects of depression on vitamin D status have been less studied, and while an earlier MR study by Milaneschi et al. did not find evidence to support a causal role [12], this may have been due to a lack of power. With the inclusion UK Biobank, and the meta-analysis with information from large-scale consortia, we were able to increase the sample size over four-fold, confirming the causal role of depression and lower 25(OH)D concentrations.

A true effect of depression on 25(OH)D concentrations is plausible and may be related to many factors. Depression is often linked to fatigue, isolation and a sedentary lifestyle, which could all translate to more time spent indoors, leading to lower exposure to sunlight. Poor dietary choices, or reduced appetite, may decrease dietary vitamin D intake, while poor diet, and lack of physical activity may also increase the risk of obesity which also has a causal effect on lowering 25(OH)D concentrations [33]. Metabolic demand for vitamin D may also be increased in those with depression to counter the imbalances in calcium homeostasis associated with this condition [1], which may in turn contribute to 25(OH)D deficiency. In support of related influences on the association between depression and 25(OH)D, adjustments for basic, socioeconomic and lifestyle factors more than halved the association in our observational analyses.

Our findings may have some clinical implications. For example, depression has been associated with low bone mineral density [34] and an increased risk of osteoarthritis [35]. It is plausible that this adverse association between depression and poor bone health may result from low 25(OH)D concentrations since low 25(OH)D can trigger a compensatory rise in levels of parathyroid hormone (PTH) [36], which not only promotes the conversion of 25(OH)D to its active form, calcitriol, but also increases bone resorption [37]. Indeed, we have recently reported a causal effect of depression on risk of osteoarthritis using the MR approach, highlighting the need to monitor bone health in people with depression [36]. Individuals with depression are also more vulnerable to respiratory infections [38], and this risk may be modifiable by regulating vitamin D levels; lower 25(OH)D concentrations have been causally linked to greater risk of bacterial pneumonias [39], and calcitriol plays a role in modulating the immune response to respiratory viruses [40]. Furthermore, RCTs indicate that the risk of acute respiratory infections can be reduced by daily or weekly vitamin D supplementation [41]. The strengths of this study include the large population size and the breadth of individual data available for participants in the UK Biobank cohort. The use of the MR approach has allowed us to assess bi-directional causality between 25(OH)D and depression. Genetic markers of 25(OH)D that have arisen from genome-wide association studies are useful instruments for MR analyses of 25(OH)D [42] and we have previously utilized this method to assess the causal relationships between vitamin D status and obesity [33], blood pressure [43], and cognitive function [44]. Similarly, we have used genetically instrumented depression scores to investigate causal effects of depression on BMI [18], and multiple disease outcomes [35]. Although RCT is the gold standard for testing of causality, the MR will help us to avoid methodological problems related to confounding and reverse causality affecting other types of observational studies. Furthermore, this approach allowed us to test the causal link between lifetime nutritional vitamin D status and depression, which most intervention studies struggle to achieve, and which is sometimes mentioned as one possible reason for mixed results from RCT [45]. Mixed results from RCTs could also be due to methodological flaws such as lack of baseline 25(OH)D measures (preventing the ability to demonstrate a change in concentrations), interventions that result in no change in 25(OH)D concentrations, or reduced rather than increased concentrations, and starting with baseline 25(OH)D levels that indicated sufficiency (not deficiency) [7]. These factors were noted as potential methodological flaws with earlier studies, with evidence for differential conclusions in higher and lower quality studies [7].

There are further methodological considerations with our approach. While we did not detect evidence for a causal role of 25(OH)D in depression, we cannot rule out the possibility that lower 25(OH)D makes a small contribution to depression risk. In the combined UK Biobank consortia meta-analyses (*n* = 424,967), our study was powered to detect the OR of 0.92 per 50% higher serum 25(OH)D, which is a slightly stronger association than that suggested by the observational data (0.95, 0.94 to 0.96) or MR analyses (0.97, 0.93 to 1.02). Furthermore, while our results argue against linear increases in 25(OH)D having a substantial influence on depression, we may not have been able to detect effects of 25(OH)D ‘deficiency’ with this approach since the instrument captures differences in average 25(OH)D across the continuum and assumes linear effects. However, given complex biology and the heterogeneity across the different types of ‘depression’, further studies are warranted to establish more specific associations between 25(OH)D and, for example, seasonal affective disorder. Although we included self-report and hospital inpatient information, the definition of depression was not gold standard due to lack of a valid depression diagnostic instruments in the UK Biobank [46]. With only 5% participation rate and some selection towards relatively healthy participants [47], results from the UK Biobank may be prone to collider bias if participation to the study is affected by serum 25(OH)D and depression status. However, collider bias is unlikely to have had a substantial effect on findings as the estimates were remarkably consistent also when using summary data from consortia that did not include the UK Biobank. As the current study is restricted to white British participants, the findings of this study may not be representative of other populations. Finally, despite efforts to include various MR methods that rely on different pleiotropic assumptions, we cannot fully rule out bias in the causal estimates due to horizontal pleiotropic effects (a situation where the genetic instruments associate with the outcome through pathways other than through the exposure).

In conclusion, we provided genetic evidence that depression contributes to low 25(OH)D concentrations but found little evidence for a material contribution by vitamin D status on the risk of depression. Our study suggests that while vitamin D may not help to prevent depression, monitoring and treatment of vitamin D deficiency may be beneficial in alleviating adverse influences of depression on health.

## Figures and Tables

**Table 1:** Prevalence of depression and summary of serum 25(OH)D (in nanomoles per litre, nmol/L) across different characteristics.

	*n* (%)	Depression		Serum 25(OH)D in nmol/L	
		*n* (%)	*p*-Value ^1^	Median (IQR)	*p*-Value ^2^
Sex			<1.0 × 10^−300^		0.02
Male	116,698 (50.8)	11,292 (9.7)		48.8 (34.4, 64.0)	
Female	113,134 (49.2)	18,855 (16.7)		48.9 (34.3, 63.9)	
Age			7.7 × 10^−87^		<1.0 × 10^−300^
39–49 years	51,191 (22.3)	7201 (14.1)		46.1 (32.1, 62.0)	
50–59 years	73,641 (32.0)	10,548 (14.3)		47.5 (33.2, 62.8)	
60–73 years	105,000 (45.7)	12,398 (11.8)		51.0 (36.6, 65.4)	
BMI			4.5 × 10^−154^		<1.0 × 10^−300^
Underweight, <18.5 kg/m^2^	1083 (0.5)	167 (15.4)		48.1 (31.2, 67.2)	
Normal, (≥18.5 and <25) kg/m^2^	75,087 (32.7)	9031 (12.0)		52.3 (36.7, 67.6)	
Overweight, (≥25 and <30) kg/m^2^	98,778 (43.0)	11,961 (12.1)		49.8 (35.6, 64.3)	
Obese, ≥30 kg/m^2^	54,151 (23.6)	8821 (16.3)		42.9 (30.2, 57.2)	
Missing	733 (0.3)	167 (22.8)		39.7 (25.9, 56.0)	
Education			3.4 × 10^−13^		6.0 × 10^−66^
None	38,458 (16.7)	5030 (13.1)		49.7 (34.8, 65.0)	
NVQ/CSE/A levels	81,147 (35.3)	11,044 (13.6)		49.7 (35.0, 64.9)	
Degree/professional	108,287 (47.1)	13,881 (12.8)		47.9 (33.8, 62.8)	
Missing	1940 (0.8)	192 (9.9)		50.0 (34.7, 65.0)	
Physical activity			2.0 × 10^−200^		<1.0 × 10^−300^
None	12,000 (5.2)	2542 (21.2)		35.4 (23.9, 51.7)	
Light/moderate	191,144 (83.2)	25,075 (13.1)		48.9 (34.7, 63.7)	
Strenuous sports	25,932 (11.3)	2337 (9.0)		54.2 (39.3, 69.2)	
Missing	756 (0.3)	193 (25.5)		38.6 (24.7, 56.1)	
Oily fish consumption			4.8 × 10^−36^		<1.0 × 10^−300^
Never	24,100 (10.5)	3775 (15.7)		44.2 (29.7, 60.6)	
<Once a week	76,738 (33.4)	10,090 (13.2)		47.1 (32.6, 62.6)	
Once a week	87,870 (38.2)	10,737 (12.2)		49.9 (35.7, 64.6)	
>Once a week	40,130 (17.5)	5400 (13.5)		52.2 (38.2, 66.4)	
Missing	994 (0.4)	145 (14.6)		45.9 (30.6, 61.9)	
Sun protection use			1.8 × 10^−60^		<1.0 × 10^−300^
Do not go in sunshine	1048 (0.5)	251 (24.0)		31.7 (21.1, 45.7)	
Never/rarely	18,794 (8.2)	2506 (13.3)		43.9 (29.8, 59.8)	
Sometimes	77,559 (33.7)	9443 (12.2)		48.6 (34.3, 63.5)	
Most of the time	84,409 (36.7)	11,164 (13.2)		49.7 (35.4, 64.6)	
Always	47,902 (20.8)	6764 (14.1)		49.7 (34.9, 65.0)	
Missing	120 (0.1)	19 (15.8)		33.2 (22.4, 48.6)	
Long standing illness			<1.0 × 10^−300^		5.9 × 10^−280^
No	156,658 (68.2)	15,275 (9.8)		49.7 (35.3, 64.5)	
Yes	68,590 (29.8)	14,113 (20.6)		46.8 (32.2, 62.6)	
Missing	4584 (2.0)	759 (16.6)		46.9 (32.9, 61.9)	

^1^*p*-value from likelihood ratio test in logistic regression model adjusted for adjusted for sex, age, assessment centre, and date of blood sample collected. ^2^*p*-value from likelihood ratio test in linear regression model adjusted for sex, age, assessment centre, and date of blood sample collected. See Table S3 for more list of covariates.

**Table 2:** Association between serum 25(OH)D and depression.

	*n* (%)	Depression *n* (%)	Odds of Depression (*n* = 202,413) ^6^		
			Basic ^1^ OR (95%CI)	Socioeconomic ^2^ OR (95%CI)	Lifestyle ^3^ OR (95%CI)
Serum 25(OH)D level ^4^					
<25	21,688 (10.7)	3209 (14.8)	Reference	Reference	Reference
≥25 and <50	82,389 (40.7)	10,548 (12.8)	0.72 (0.68, 0.75)	0.76 (0.73, 0.80)	0.85 (0.81, 0.89)
≥50 and <75	72,843 (36.0)	9206 (12.6)	0.64 (0.61, 0.67)	0.70 (0.66, 0.73)	0.83 (0.79, 0.87)
>75	25,493 (12.6)	3312 (13.0)	0.62 (0.58, 0.66)	0.67 (0.63, 0.71)	0.83 (0.78, 0.88)
Per 50% higher serum 25(OH)D ^5^	202,413	26,270 (13.0)	0.87 (0.86, 0.89)	0.90 (0.88, 0.91)	0.95 (0.94, 0.96)
P_trend_			2.1 × 10^−72^	4.9 × 10^−50^	4.0 × 10^−12^
P_curvature_			8.0 × 10^−12^	2.4 × 10^−6^	2.8 × 10^−4^
P_sex-interaction_			3.8 × 10^−6^	7.9 × 10^−4^	9.4 × 10^−4^
P_age-interaction_			0.03	0.04	0.07

^1^ Basic model included adjustment for basic covariates including age, sex, assessment centre, and date of blood sample collected. ^2^ Socioeconomic model included adjustment for basic and socioeconomic-related covariates including education, Townsend deprivation index, and employment. ^3^ Lifestyle model included adjustment for basic, socioeconomic and lifestyle-related covariates including smoking, alcohol consumption, BMI, physical activity, fish and cheese consumptions, dietary restriction, sun exposure [in summer or winter], use of sun protection and long-standing illness. ^4^ Serum 25(OH)D level expressed in nanomoles per litres (nmol/L) unit. ^5^ Natural log-transformed 25(OH)D, and effect estimates transformed to reflect per 50% higher in 25(OH)D. ^6^ Number of individuals in the complete case analyses.

## Data Availability

All data is available through the UK Biobank repository on application.

## Supplementary Materials

The following are available online at https://www.mdpi.com/2072-6643/13/1/109/s1. Supplementary note. Mendelian randomization. Table S1. List of 25(OH)D-related variants, and its association with serum 25(OH)D level among UK Biobank and discovery cohort. Table S2. List of major depressive disorder-related variants used to construct the genetic risk scores. Table S3. Prevalence of depression and summary of 25(OH)D across different characteristics. Table S4. Association between serum 25(OH)D level and depression among men and women. Table S5. Association between serum 25(OH)D and depression excluding serum 25(OH)D data from aliquot three blood sample. Table S6. The causal estimates for the association between 25(OH)D and depression using two, four and all 25(OH)D variants as the instrument in the two-sample MR analysis. Figure S1. Flow of UK Biobank participants included in the bi-directional analysis between serum 25(OH)D and depression. Figure S2. Association between MDD-related variants and depression in UK Biobank versus discovery GWAS. Figure S3. Comparison of randomized control trial (RCT) with Mendelian randomization (MR) [16] [Panel A], and summary of analyses strategy [Panel B]. Figure S4. Genetic instrument validation. Plot A shows the distribution of 25(OH)D genetic risk score (GRS), and its association with 25(OH)D in UK Biobank, with the weighted GRS explains 2.7% of the variability in 25(OH)D. Plot B shows the association between GRS in ten-quantiles and depression in UK Biobank, with the weighted GRS explains 0.2% of the variability in the depression. Figure S5. Plots from two-sample MR analysis of 25(OH)D on depression. Plot A shows the Scatter plot of the estimates of variant-depression association against estimates of variant-25(OH)D association. Plot B shows the funnel plots of instrument strength against causal estimate (βIV). Plot C includes leave-one-out analyses, demonstrating the effect on the overall MR IVW estimate by excluding each of the six variants one at a time. Figure S6. Observational and MR analyses on the association between 25(OH)D (using the 122 25(OH)D-related variants from Revez et al. [26]) and the odds of depression. Figure S7. Plots from two-sample MR analysis of depression on 25(OH)D. Plot A shows the scatter plot of the estimates of variant-25(OH)D association against estimates of variant-depression association. Plot B shows the funnel plots of instrument strength against causal estimate (βIV). Plot C includes leave-one-out analyses, demonstrating the effect on the overall MR IVW estimate by excluding each of the 44 variants one at a time. Figure S8. Two-sample MR estimates from different MR approaches using 17 major depression-related genetic variants from Hyde et al. [29]. MR Egger intercept *p*-value = 0.87. Figure S9. Percent change in serum 25(OH)D associated with depression (Observational), or genetic liability to depression (MR) using 17 major depression-related genetic variants from Hyde et al. [29].
