# Supplementary material for: Relationship between Serum 25(OH)D and Depression: Causal Evidence from a Bi-Directional Mendelian Randomization Study
Source: Nutrients. 2020 Dec 30;13(1):109. doi: 10.3390/nu13010109 (PMC7823924; doi:10.3390/nu13010109)
Supplement: Supplementary file 1 [file nutrients-13-00109-s001.pdf]

Supplementary note: Mendelian Randomisation

Mendelian randomisation (MR) uses genetic variants (commonly from genome-wide association studies (GWAS) as a proxy measure ("genetic instrument") for the exposure in the hypothesis testing of the causal association of the exposure on the outcome (1).Unlike observational association studies, MR studies are less affected by unmeasured confounding factors and reverse causation as the genetic variants are defined at the time of conception (2). Commonly, MR referred as the natural randomised control trial (2) (Figure S3 Panel A). In our study, we selected the genetic variants that instrument the exposure of interest (25(OH)D or depression, depending on the direction of the association) from the latest GWAS (preferably not including UK Biobank, unless it is for sensitivity analysis), and applied different MR methods (Inverse variance weight, MR-PRESSO, weighted median, weighted mode and MR-Egger) for testing the bidirectional causal association between 25(OH)D and depression. The coefficients from MR analyses may indicate a valid causal effect estimate under the condition of the following three core assumptions: the genetic instrument needs to be robustly associated with the exposure ('relevance'), there should be no joint causal influence affecting both the instrument and the outcome ('independence'), and the instrument should not affect the outcome through any other mechanism than through the exposure ('exclusion restriction') (2). Various analyses including MR-PRESSO outlier detection and distortion tests, MR-Egger intercept test, and leave-one-out analyses were applied to check for the effect of horizontal pleiotropy (violation of 'exclusion restriction' assumption) and restricted the analyses to white British with further adjustment for 40 principal components and assessment centre for controlling residual confounding effect from population structure (violation of 'independence' assumption). Commonly the first assumption ('relevance') fulfilled when variants, that are associated with the exposure at genome-wide significant level (5×10^{-8}), are taken from the GWAS.

Table S1. List of 25(OH)D-related variants, and its association with serum 25(OH)D level among UK Biobank and discovery cohort

				UK Biobank					Discovery GWAS(3)			
SNP	Chr.	Nearest Gene	EA/OA	MAF	Info Score	Beta	SE	*p*-Value	MAF	Beta	SE	P
rs3755967	4	GC	C/T	0.29	0.99	0.0840	0.0014	2.6E-365	0.28	0.0892	0.0023	4.7E-343
rs12785878	11	DHCR7	T/G	0.21	1.00	0.0521	0.0016	5.00E-236	0.25	0.0363	0.0022	3.81E-62
rs10741657	11	CYP2R1	A/G	0.41	1.00	0.0351	0.0013	7.00E-225	0.40	0.0308	0.0022	2.05E-46
rs10745742	12	AMDHD1	T/C	0.38	0.99	0.0128	0.0014	1.10E-21	0.40	0.0165	0.0022	1.88E-14
rs8018720	14	SEC23A	G/C	0.18	1.00	0.0151	0.0017	1.20E-27	0.18	0.0168	0.0029	4.72E-09
rs17216707	20	CYP24A1	T/C	0.18	0.96	0.0171	0.0017	2.00E-25	0.21	0.0263	0.0027	8.14E-23

SNP: single nucleotide polymorphism; EA/OA: effect allele/other allele; MAF: Minor allele frequency. Info score (imputation quality indicator, all with >0.96 info score). Effect estimates from UK Biobank and discovery GWAS were from linear regression of variants on natural-log transformed 25(OH)D. No genetic overlap with variants used to index depression (Correlation $R^2 < 0.00386$ for all).

Table S2. List of major depressive disorder-related variants used to construct the genetic risk scores

The 44 major depressive disorder-related variants from Wray et al.(4)

Variant	Chr.	Nearest gene	EA/OA	MAF	INFO Score	Proxy Variant used?	Hyde et al Variant Replicated in Wray et al (r^2 with the Lead Variant of Wray et al)
rs12129573	1	*LINC01360*	A/C	0.35	0.99	No	rs2422321 (0.25)
rs1432639	1	*NEGR1*	A/C	0.40	0.99	No	rs11209948 (1.00)
rs159963	1	*RERE, SLC45A1*	C/A	0.41	0.99	No	rs301806 (0.96)
rs2389016	1		T/C	0.30	0.99	No	
rs4261101	1		G/A	0.36	0.99	No	
rs9427672	1	*DENND1B*	G/A	0.23	0.99	No	
rs11682175	2	*VRK2*	C/T	0.47	0.99	No	rs1518395 (0.38)
rs1226412	2	*LINC01876, NR4A2, GPD2*	T/C	0.20	0.99	No	
rs9862324[1]	3	*TOPAZ1, TCAIM, ZNF445*	C/T	0.32	0.99	Yes	
rs7430565	3	*RSRC1, LOC1000996447, MLF1*	G/A	0.42	0.99	No	rs1656369 (0.28)
rs34215985	4	*SLC30A9, LINC00682, DCAF4L1*	G/C	0.20	0.98	No	
rs2018142[1]	5		C/A	0.48	0.99	Yes	
rs27732[1]	5	*LINC00461, MEF2C*	A/G	0.40	0.98	Yes	rs454214 (0.89)
rs11135349	5		C/A	0.46	0.99	No	rs4543289 (0.99)
rs34660260[2]	5	*LOC101927421*	C/T	0.40	0.98	No	
rs4869056	5	*TENM2*	G/A	0.38	0.99	No	
rs3095337[2]	6	*extended MHC*	G/C	0.21	0.99	No	
rs9402472	6	*C6orf168, FBXL4*	A/G	0.24	0.98	No	
rs6460902[1]	7	*TMEM106B, VWDE*	A/G	0.42	0.99	Yes	
rs12666117	7		A/G	0.46	0.99	No	
rs958538[1]	9		T/C	0.25	0.99	Yes	
rs1354115	9	*PUM3, LINC01231*	A/C	0.37	0.99	No	rs7044150 (0.96)
rs7029033	9	*DENND1A, LHX2*	T/C	0.07	0.99	No	
rs7856424	9	*ASTN2*	C/T	0.28	0.99	No	
rs61867293	10	*SORCS3*	C/T	0.19	0.99	No	rs10786831 (0.11)
rs1806153	11	*DKFZp686K1684, PAUPAR, ELP4*	T/G	0.23	0.99	No	
rs4074723	12	*SOX5*	C/A	0.41	0.99	No	
rs4143229	13	*ENOX1, LACC1, CCDC122*	C/A	0.44	0.99	No	
rs12552	13	*OLFM4, LINC01065*	A/G	0.07	0.99	No	rs12552 (1.00)
rs3742786[1]	14	*DLST, PROX2, RPS6KL1*	A/G	0.46	0.99	Yes	
rs10149470	14	*BAG5, APOPT1*	G/A	0.48	0.99	No	
rs4904738	14	*LRFN5*	C/T	0.43	0.99	No	
rs915057	14	*SYNE2, MIR548H1, ESR2*	G/A	0.43	0.99	No	
rs8025231	15		C/A	0.45	0.99	No	rs8025231 (1.00)
rs11643192	16	*PMFBP1, DHX38*	A/C	0.38	0.99	No	
rs7198928	16	*RBFOX1*	T/C	0.38	0.98	No	
rs7200826	16	*SHISA9, CPPED1*	T/C	0.26	0.99	No	
rs8063603	16	*RBFOX1*	G/A	0.32	0.98	No	
rs17727765	17	*CRYBA1, MYO18A, NUFIP2*	C/T	0.08	0.95	No	
rs11663393	18	*DCC, MIR4528*	A/G	0.46	0.99	No	
rs12958048	18	*TCF4, MIR4529*	A/G	0.33	0.99	No	
rs1833288	18	*RAB27B, CCDC68*	A/G	0.28	0.97	No	
rs62099069	18	*MIR924HG*	T/A	0.42	0.99	No	
rs5758265	22	*L3MBTL2, EP300-AS1, CHADL*	A/G	0.29	0.99	No	rs2179744 (0.98)

The 17 major depressive disorder-related variants from Hyde et al.(5)

Variant	Chr.	Nearest gene	EA/OA	MAF	INFO score	Proxy variant used?
rs301806	1	*RERE*	C/T	0.42	0.99	No
rs2422321	1	*NEGR1*	G/A	0.43	0.98	No
rs11209948	1	*NEGR1*	T/G	0.40	1.00	No
rs12065553	1		G/A	0.30	0.99	No
rs1518395	2	*VRK2*	*G/A*	0.39	0.99	No
rs1656369	3	*RSRC1, MLF1*	*T/A*	0.35	0.99	No
rs454214	5	*TMEM161B, MEF2C*	*C/T*	0.42	0.99	No
rs4543289	5		T/G	0.45	0.99	No
rs10514299	5	*TMEM161B, MEF2C*	T/C	0.25	0.99	No
rs1475120	6	*HACE1, LIN28B*	*A/G*	0.45	0.99	No
rs6476606	9	*PAX5*	*A/G*	0.37	0.98	No
rs7044150	9	*KIAA0020, RFX3*	*T/C*	0.37	0.99	No
rs10786831	10	*SORCS3*	A/G	0.41	0.99	No
rs2125716	12	*SLC6A15*	G/A	0.23	0.99	No
rs12552	13	*OLFM4*	A/G	0.44	0.99	No
rs8025231	15	*MEIS2, TMCO5A*	C/A	0.45	0.99	No
rs2179744	22	*L3MBTL2*	A/G	0.28	0.99	No

[1] Imputation quality was poor for all six variants (info score <0.89 and MAF<0.01), hence we replaced them with a proxy variant (LD $r^2 \geq 0.8$, info score ≥ 0.95). [2] These indicate SNPs (single nucleotide polymorphism) with two alternative rs-numbers: rs116755193 for rs34660260, and rs115507122 for rs3095337. EA/OA: Effect allele/Other allele. MAF: Minor allele frequency. Please see Figure S2 for the association of these variants with MDD among UK Biobank and discovery study.

Table S3. Prevalence of depression and summary of 25(OH)D across different characteristics

	n (%)	Depression		Serum 25(OH)D in nmol/L	
		n (%)	*p*-Value[1]	Median (IQR)	*p*-Value[2]
Townsend deprivation index			3.8×10^{-105}		$<1.0 \times 10^{-300}$
• Highly deprived (above median)	113,021 (49.3)	17,640 (15.6)		46.5 (32.2, 62.1)	
• Less deprived (below median)	116,564 (50.7)	12,462 (10.7)		50.9 (36.7, 65.5)	
• Missing	247 (0.1)	45 (18.2)		47.0 (34.0, 62.1)	
Employment			$<1.0 \times 10^{-300}$		$< 1.0 \times 10^{-300}$
• No	16,140 (7.0)	4408 (27.3)		45.1 (30.3, 61.7)	
• Retired	80,489 (35.0)	10,034 (12.5)		52.4 (37.8, 66.8)	
• Lower working hour (1st quartile)	30,645 (13.3)	4269 (13.9)		49.7 (35.4, 64.2)	
• 2nd quartile working hour	21,316 (9.3)	2975 (14.0)		46.7 (32.9, 62.2)	
• 3rd quartile working hour	41,517 (18.1)	4852 (11.7)		45.7 (31.9, 61.1)	
• Higher working hour (4th quartile)	37,363 (16.3)	3322 (8.9)		46.3 (32.6, 61.5)	
• Missing	2362 (1.0)	287 (12.2)		48.6 (34.6, 63.7)	
Diet restriction			1.1×10^{-36}		1.7×10^{-65}
• No egg/diary containing food	8494 (3.7)	1355 (16.0)		47.5 (32.9, 63.4)	
• No wheat containing food	3708 (1.6)	694 (18.7)		50.3 (35.2, 66.5)	
• No sugar or sugar containing food/drink	39,733 (17.3)	4991 (12.6)		50.6 (36.0, 65.7)	
• Eat all above	177,374 (77.2)	23,040 (13.0)		48.5 (34.1, 63.5)	
• Missing	523 (0.2)	67 (12.8)		48.1 (32.2, 64.6)	
Time spend outdoor in summer (in hour)			4.1×10^{-21}		$<1.0 \times 10^{-300}$
• None	342 (0.2)	91 (26.6)		34.6 (24.2, 51.0)	
• <One	7991 (3.5)	1,360 (17.0)		40.0 (27.6, 55.6)	
• One	19,378 (8.4)	2617 (13.5)		43.9 (30.9, 59.0)	
• Two	46,720 (20.3)	6166 (13.2)		46.5 (32.5, 61.3)	
• Three	37,298 (16.2)	4975 (13.3)		48.6 (34.3, 63.3)	
• Four	35,537 (15.5)	4677 (13.2)		50.4 (36.0, 65.3)	
• Five	24,630 (10.7)	3246 (13.2)		52.1 (37.5, 66.8)	
• Six and above	46,642 (20.3)	5698 (12.2)		53.2 (38.5, 68.1)	
• Missing	11,294 (4.9)	1317 (11.7)		43.5 (29.8, 59.1)	
Time spend outdoor in winter (in hour)			3.5×10^{-18}		$<1.0 \times 10^{-300}$
• None	6326 (2.8)	1164 (18.4)		44.1 (29.3, 61.5)	
• <One	34,449 (15.0)	4725 (13.7)		45.9 (31.2, 61.9)	
• One	72,318 (31.5)	9492 (13.1)		47.4 (33.1, 62.7)	
• Two	53,254 (23.2)	6962 (13.1)		48.7 (34.2, 63.6)	
• Three	20,446 (8.9)	2727 (12.6)		49.5 (35.3, 64.3)	
• Four	13,580 (5.9)	1711 (12.6)		50.0 (35.6, 65.0)	
• Five	6325 (2.8)	784 (12.4)		50.1 (35.3, 65.0)	

•	Six and above	11,691 (5.1)	1233 (10.6)		49.6 (35.3, 65.3)	
•	Missing	11,443 (5.0)	1349 (11.8)		45.9 (31.1, 61.9)	
Non-oily fish consumption				2.8×10^{-24}		8.8×10^{-219}
•	Never	9300 (4.1)	1520 (16.3)		42.9 (28.3, 59.7)	
•	<Once a week	65,158 (28.4)	8889 (13.6)		47.6 (33.0, 63.0)	
•	Once a week	117,116 (51.0)	14,478 (12.4)		49.5 (35.2, 64.4)	
•	>Once a week	37,521 (16.3)	5159 (13.8)		50.1 (35.9, 65.1)	
•	Missing	737 (0.3)	101 (13.7)		45.7 (31.0, 60.8)	
Cheese consumption				2.3×10^{-11}		2.2×10^{-60}
•	Never	5717 (2.5)	817 (14.3)		48.8 (33.6, 64.2)	
•	<Once a week	35,525 (15.5)	5174 (14.6)		49.6 (35.0, 64.8)	
•	Once a week	47,776 (20.8)	6143 (12.9)		49.5 (34.9, 64.7)	
•	>Once a week	135,724 (59.1)	17,154 (12.6)		48.4 (34.0, 63.4)	
•	Missing	5090 (2.2)	859 (16.9)		48.6 (33.8, 63.7)	

[1] p-value from likelihood ratio test in logistic regression model adjusted for sex, age, assessment centre, and date of blood sample collected. [2] p-value from likelihood ratio test in linear regression model adjusted for sex, age, assessment centre, and date of blood sample collected.

	Men (n = 104,257)				Women (n = 98,156)			
	Depression n(%)	Basic[1] OR (95%CI)	Socio-economic[2] OR (95%CI)	Lifestyle[3] OR (95%CI)	Depression n(%)	Basic[1] OR (95%CI)	Socioeconomic[2] OR (95%CI)	Lifestyle[3] OR (95%CI)
Serum 25(OH)D level[4]								
• <25	1266 (11.4)	Reference	Reference	Reference	1943 (18.4)	Reference	Reference	Reference
• ≥25 and <50	4034 (9.5)	0.68 (0.64, 0.73)	0.76 (0.71, 0.82)	0.85 (0.78, 0.91)	6514 (16.4)	0.74 (0.70, 0.79)	0.77 (0.73, 0.83)	0.86 (0.81, 0.92)
• ≥50 and <75	3381 (9.0)	0.57 (0.53, 0.61)	0.66 (0.61, 0.71)	0.78 (0.72, 0.85)	5820 (16.5)	0.68 (0.64, 0.73)	0.74 (0.69, 0.79)	0.88 (0.82, 0.94)
• ≥75	1232 (9.4)	0.55 (0.50, 0.61)	0.63 (0.58, 0.70)	0.78 (0.71, 0.86)	2080 (16.7)	0.66 (0.61, 0.72)	0.71 (0.66, 0.77)	0.88 (0.81, 0.96)
Per 50% higher serum 25(OH)D[5]	9913 (9.5)	0.83 (0.82, 0.85)	0.87 (0.85, 0.89)	0.92 (0.89, 0.94)	16,357 (16.7)	0.89 (0.88, 0.91)	0.91 (0.90, 0.93)	0.98 (0.95, 0.99)
P_{trend}		1.3×10^{-76}	5.5×10^{-41}	1.3×10^{-13}		1.9×10^{-42}	4.2×10^{-26}	0.02
$P_{curvature}$		1.2×10^{-6}	0.01	0.04		3.8×10^{-6}	3.5×10^{-4}	0.008

Table S4. Association between serum 25(OH)D level and depression among men and women.

[1] Basic model included adjustment for basic covariates including age, assessment centre, and date of blood sample collected.[2] Socioeconomic model included adjustment for basic and socioeconomic-related covariates including education, Townsend deprivation index, and employment.[3] Lifestyle model included adjustment for basic, socioeconomic and lifestyle-related covariates including long standing illness, BMI, physical activity, fish and cheese consumptions, sun exposure [at summer or winter], and use of sun protection. [4] Serum 25(OH)D level expressed in nanomole/litre (nmol/L) unit. [5] natural-log transformed 25(OH)D, and effect estimates transformed to reflect per 50% higher in 25(OH)D

Table S5. Association between serum 25(OH)D and depression excluding serum 25(OH)D data from aliquot three blood sample.

	n(%)	Depression n(%)	Odds of Depression (n = 197,921)		
			Basic[1] OR (95% CI)	Socio-economic[2] OR (95% CI)	Lifestyle[3] OR (95% CI)
Serum 25(OH)D level[4]					
<25	21,230 (10.7)	3149 (14.8)	Reference	Reference	Reference
≥25 and <50	80,581 (40.7)	10,345 (12.8)	0.72 (0.68, 0.77)	0.77 (0.73, 0.80)	0.85 (0.81, 0.89)
≥50 and <75	71,146 (36.0)	8992 (12.6)	0.64 (0.60, 0.67)	0.70 (0.66, 0.73)	0.83 (0.79, 0.88)
≥75	24,964 (12.6)	3247 (13.0)	0.62 (0.58, 0.66)	0.67 (0.63, 0.71)	0.83 (0.78, 0.89)
Per 50% higher serum 25(OH)D[5]	197,921	25,733 (13.0)	0.87 (0.86, 0.89)	0.90 (0.88, 0.91)	0.95 (0.94, 0.96)
P_{trend}			2.1×10^{-72}	4.9×10^{-50}	4.3×10^{-12}
$P_{curvature}$			2.4×10^{-11}	4.3×10^{-6}	3.8×10^{-4}
$P_{sex\text{-}interaction}$			3.3×10^{-6}	6.5×10^{-4}	7.3×10^{-4}
$P_{age\text{-}interaction}$			0.02	0.03	0.05

[1] Basic model included adjustment for basic covariates including age, sex, assessment centre, and date of blood sample collected. [2] Socio-economic model included adjustment for basic and socioeconomic-related covariates including education, Townsend deprivation index, and employment. [3] Lifestyle model included adjustment for basic, socioeconomic and lifestyle-related covariates including long standing illness, BMI, physical activity, fish and cheese consumptions, sun exposure [at summer or winter], and use of sun protection. [4] Serum 25(OH)D level expressed in nanomoles per litres (nmol/L) unit. [5] Natural-log transformed 25(OH)D, and effect estimates transformed to reflect per 50% higher in 25(OH)D

Table S6. The causal estimates for the association between 25(OH)D and depression using two, four and all 25(OH)D variants as the instrument in the two-sample MR analysis

			Odds ratio per 50% higher in serum25(OH)D					
Exposure	Outcome	Method	OR	LCI	UCI	P	#SNP	Loci included in the analysis
25(OH)D	Depression	Inverse variance weighted	1.06	0.89	1.26	0.52	2	DHCR7 and CYP2R1
25(OH)D	Depression	Inverse variance weighted	0.96	0.88	1.04	0.30	4	GC,AMDHD1,SEC23A,and CYP24A1
25(OH)D	Depression	Inverse variance weighted	0.97	0.90	1.05	0.52	6	DHCR7, CYP2R1,GC,AMDHD1,SEC23A,and CYP24A1

Variant-25(OH)D association estimates was taken from Jiang et al GWAS (3) and the variant-depression association estimates were from analysis in the UK Biobank. #SNP: number of SNP included in the analysis

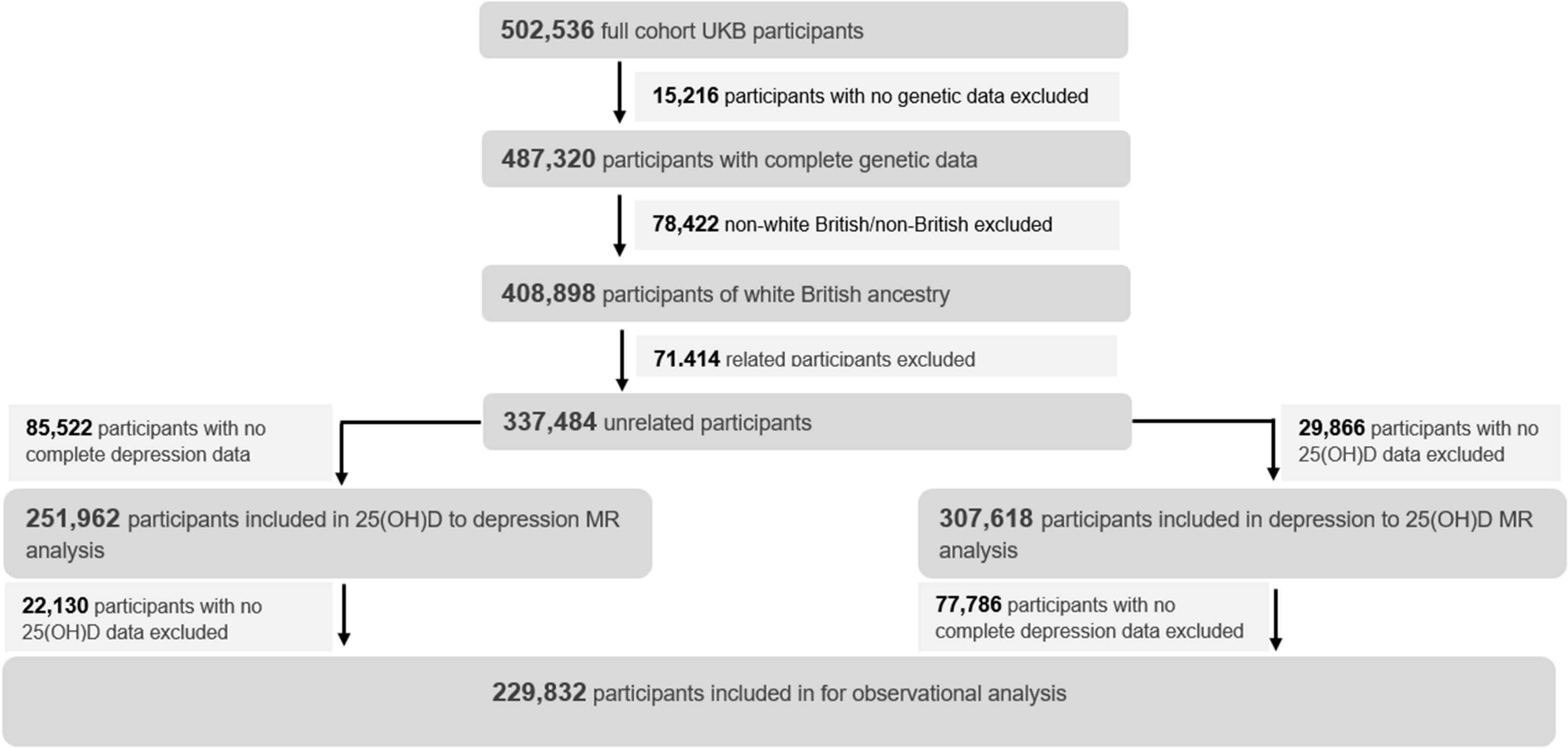

Figure S1. Flow of UK Biobank participants included in the bi-directional analysis between serum 25(OH)D and depression.

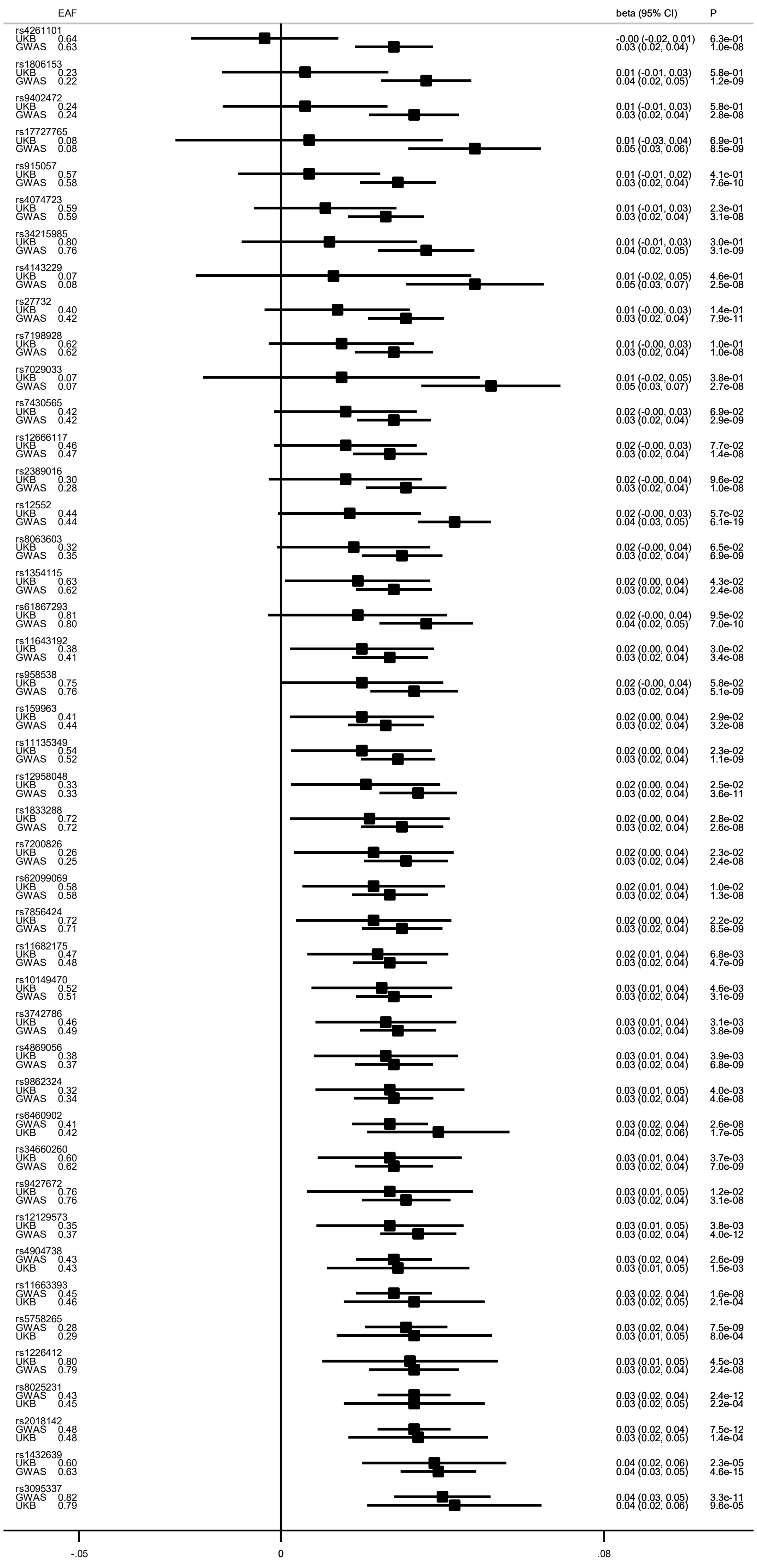

Figure S2. Association between MDD-related variants and depression in UK Biobank versus discovery GWAS.

Panel A

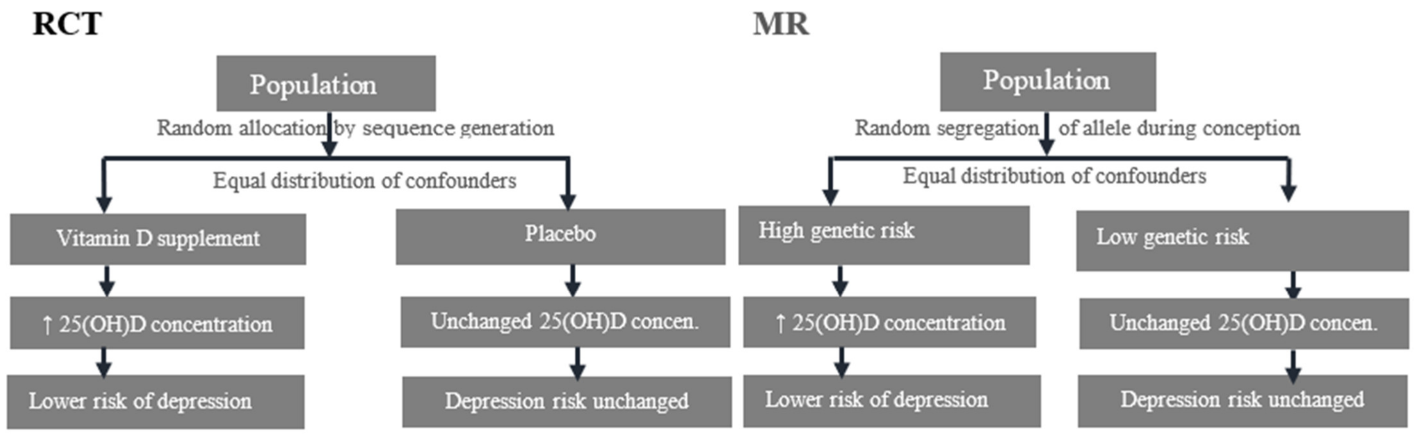

Panel B

Analytical strategy	25(OH)D ⟶ Depression	Depression ⟶ 25(OH)D
**Observational association**		
Data	UK Biobank	UK Biobank
Adjustment models		
Model-1	Age, sex, assessment center, and blood sample collection date	Age, sex, assessment center, and blood sample collection date
Model-2	Model-1 + Townsend deprivation, education, and employment	Model-1 + Townsend deprivation, education, and employment
Model-3	Model-1 + Model-2 + smoking, alcohol consumption, BMI, physical activity, sun exposure in summer and winter, use of sun protection, diet restriction, fish and cheese consumption, and long standing illness	Model-1 + Model-2 + smoking, alcohol consumption, BMI, physical activity, sun exposure in summer and winter, use of sun protection, diet restriction, fish and cheese consumption, and long standing illness
**Mendelian randomisation**		
Data	UK Biobank and Consortia	UK Biobank and Consortia
Genetic instrument	Six 25(OH)-related variants from Jiang *et al.* GWAS (main analysis)<br><br>122 25(OH)D-related variants from Revez *et al.* GWAS which is based on UK Biobank (sensitivity analysis)	44 major depressive disorder (MDD)-related variants from Wray *et al.* GWAS (main analysis)<br>17 MDD-related variants from Hyde *et al.* GWAS (sensitivity analysis)
Variant-exposure estimates	From Jiang et al. or Revez *et al.* GWAS depending on the instrument	From Wray et al. or Hyde *et al.* GWAS depending on the instrument
Variant-outcome estimates	From UK Biobank (from model adjusted for age, sex, assessment centre, 40 principal components and genotyping array)<br>From Consortia (Wray *et al.* GWAS summary reports)	From UK Biobank (from model adjusted for age, sex, assessment centre, 40 principal components and genotyping array)<br>From consortia (Jiang *et al.* GWAS summary reports)
MR-methods used	Inverse variance weight (MR IVW)<br>MR-PRESSO<br>Weighted median<br>Weighted mode<br>MR-Egger	Inverse variance weight (MR IVW)<br>MR-PRESSO<br>Weighted median<br>Weighted mode<br>MR-Egger
Pleiotropy test	MR-Egger p-intercept<br>MR-PRESSO outlier and distortion tests<br>Leave-one-out analysis	MR-Egger p-intercept<br>MR-PRESSO outlier and distortion tests<br>Leave-one-out analysis

Figure S3. Comparison of randomized control trial (RCT) with Mendelian randomization (MR) (2) (**Panel A**), and Summary of analyses strategy (**Panel B**).

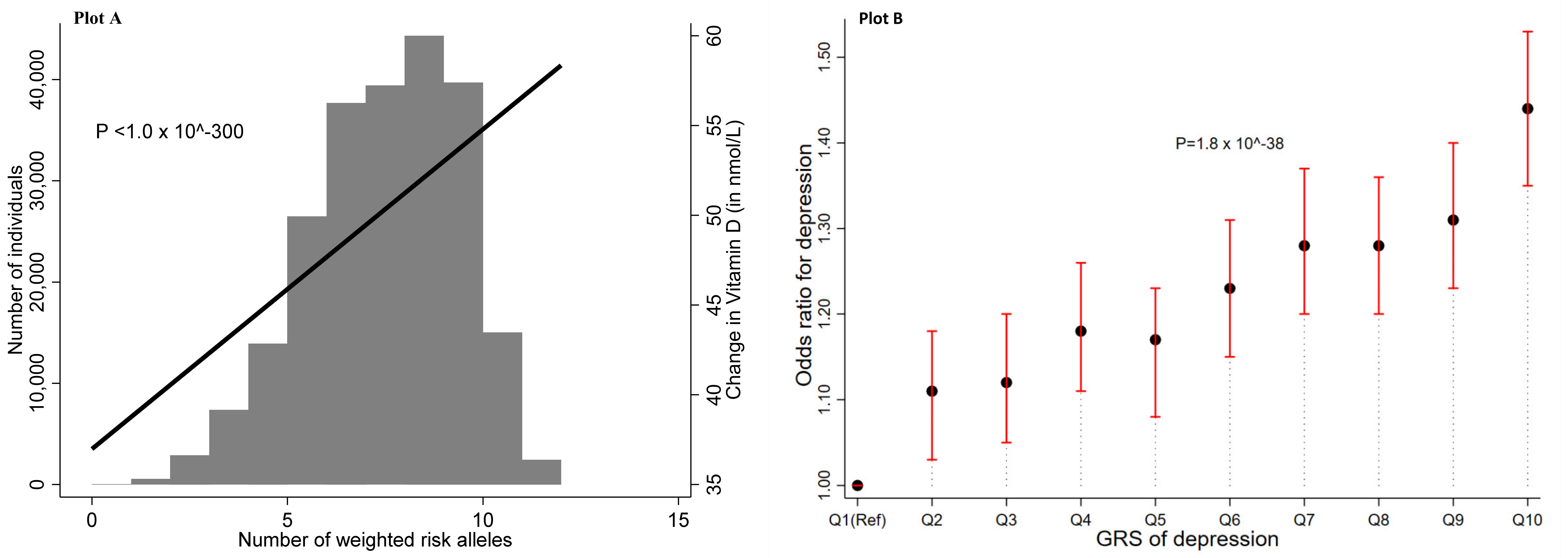

Figure S4. Genetic instrument validation. **Plot A** shows the distribution of 25(OH)D genetic risk score (GRS), and its association with 25(OH)D in UK Biobank, with the weighted GRS explains 2.7% of the variability in 25(OH)D. **Plot B** shows the association between GRS in ten-quantiles and depression in UK Biobank, with the weighted GRS explains 0.2% of the variability in the depression.

25(OH)D on Depression

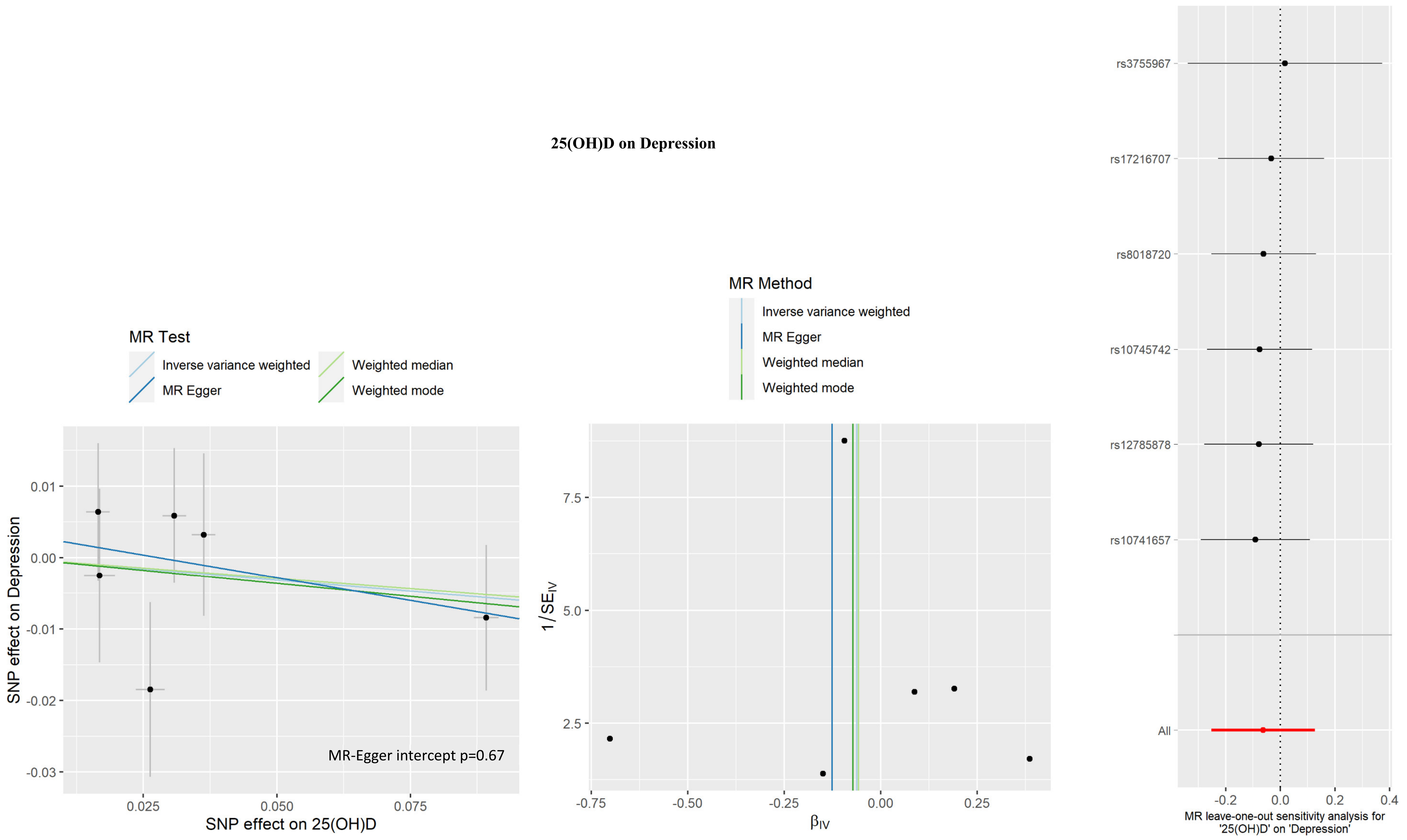

Plot A **Plot B** **Plot C**

Figure S5. Plots from two-sample MR analysis of 25(OH)D on depression. **Plot A** shows the Scatter plot of the estimates of variant-depression association against estimates of variant-25(OH)D association. **Plot B** shows the funnel plots of instrument strength against causal estimate (βIV). **Plot C** includes leave-one-out analyses, demonstrating the effect on the overall MR IVW estimate by excluding each of the six variants one at a time.

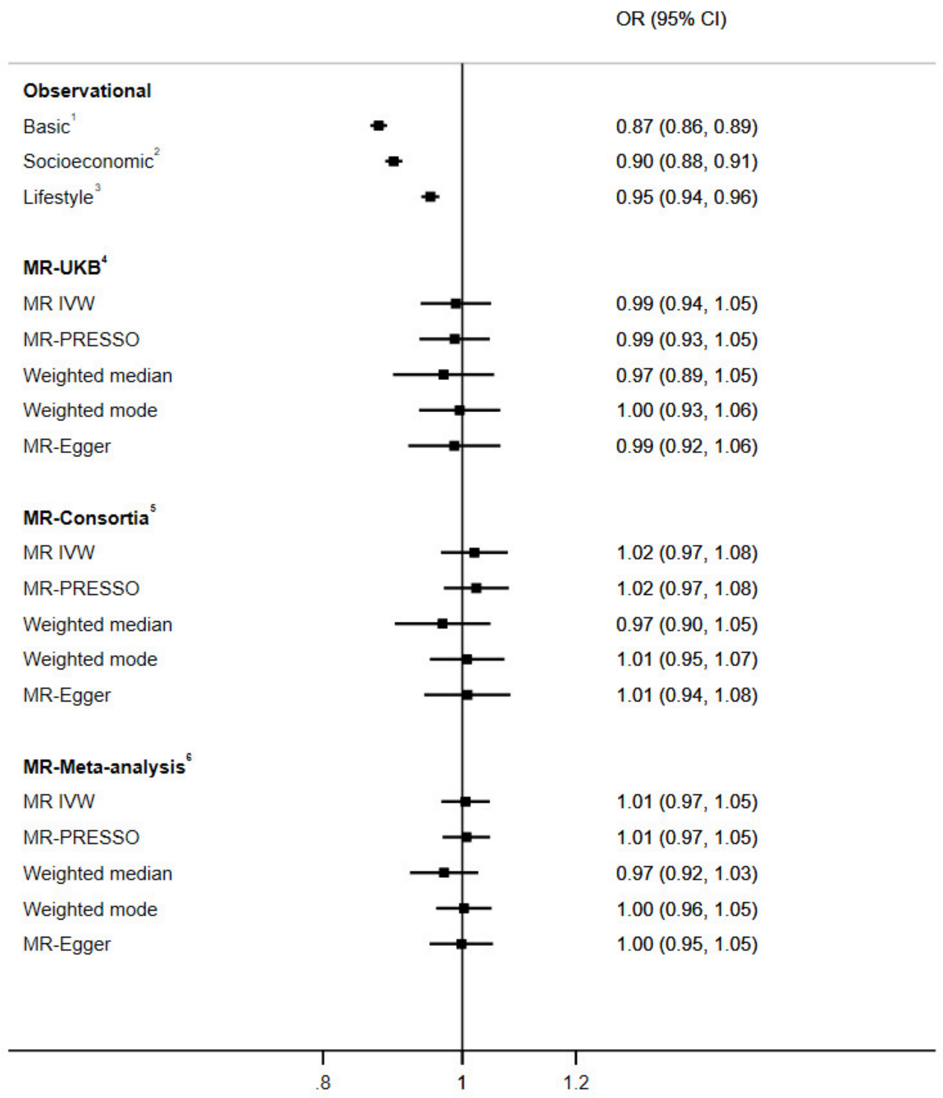

Figure S6. Observational and MR analyses on the association between 25(OH)D (using the 122 25(OH)D-related variants from Revez et al (6)) and the odds of depression. [1] Basic model included adjustment for basic covariates including age, sex, assessment center, and date of blood sample collected. [2] Socioeconomic model included adjustment for basic and socioeconomic-related covariates including education, Townsend deprivation index, and employment. [3] Lifestyle model included adjustment for basic, socioeconomic and lifestyle-related covariates including smoking, alcohol consumption, BMI, physical activity, fish and cheese consumptions, dietary restriction, sun exposure [in summer or winter], use of sun protection, and long standing illness. [4] MR analysis based on variant-depression association estimates from UK Biobank. [5] MR-analysis based on variant-depression association estimates from Wray et al GWAS. [6] Meta-analysis of MR estimates from UK Biobank and Wray et al GWAS. For all MR analysis, variant-25(OH)D estimates were from UK Biobank, subsetting the analyse to participants with no depression (Control) to minimise the bias from sample overlap. MR-Egger P-intercept (for all), $p < 0.62$.

Depression on 25(OH) D

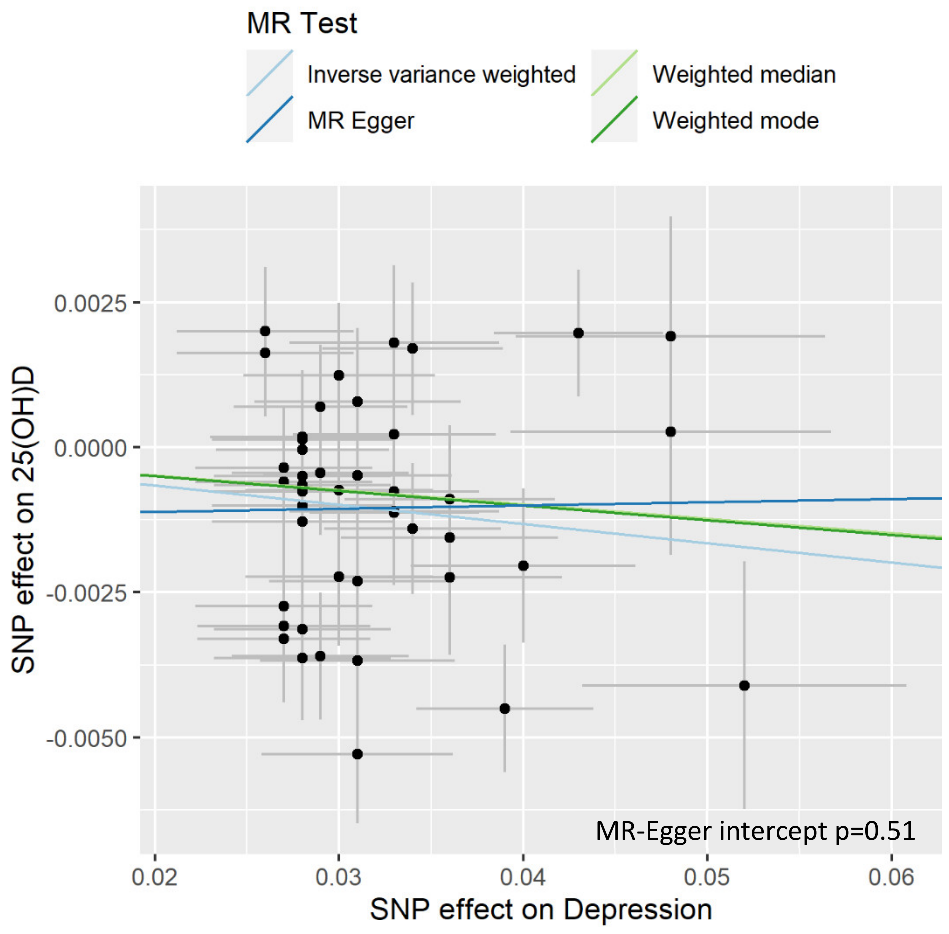

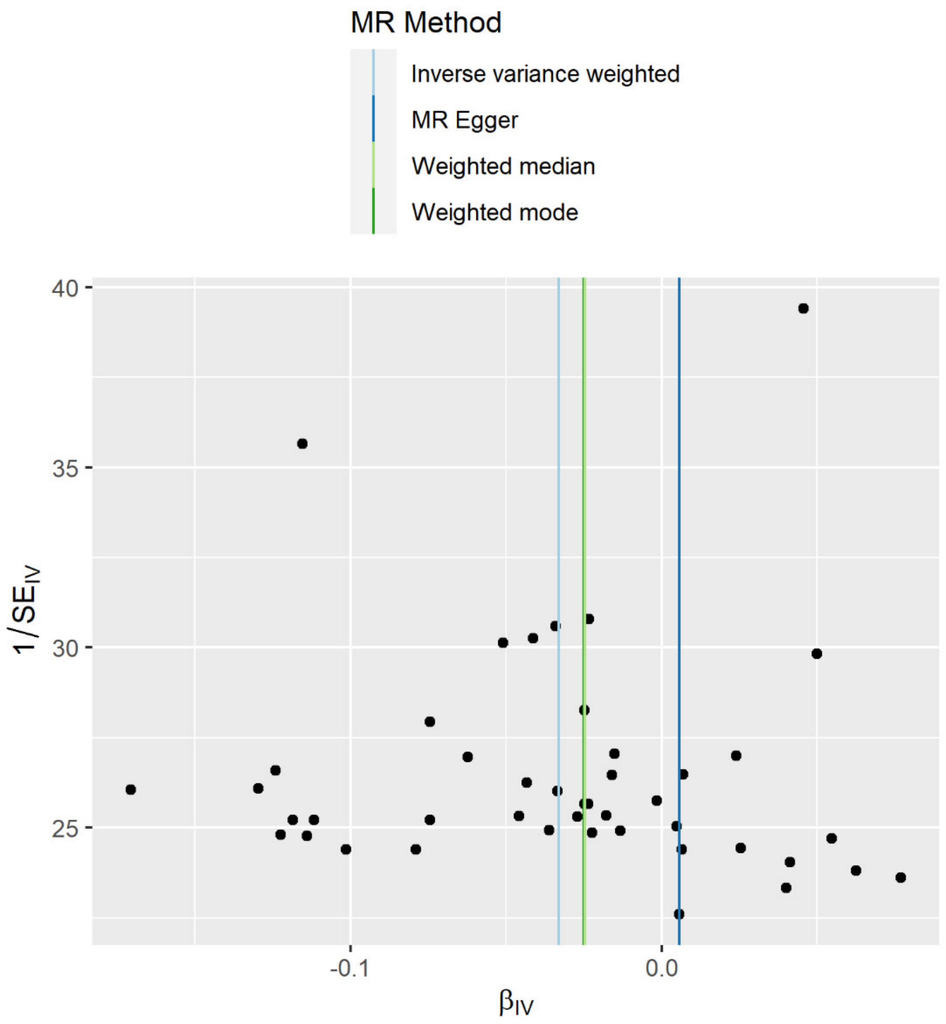

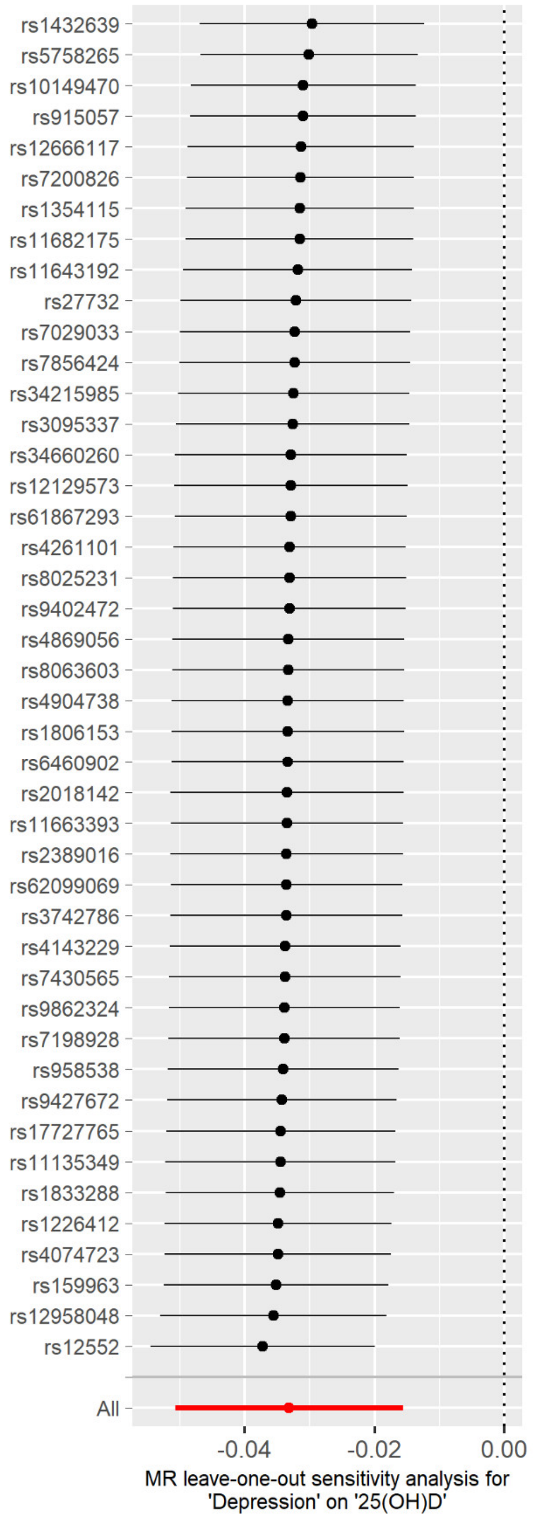

Plot A **Plot B** **Plot C**

Figure S7. Plots from two-sample MR analysis of depression on 25(OH)D. **Plot A** shows the scatter plot of the estimates of variant-25(OH)D association against estimates of variant-depression association. **Plot B** shows the funnel plots of instrument strength against causal estimate (βIV). **Plot C** includes leave-one-out analyses, demonstrating the effect on the overall MR IVW estimate by excluding each of the 44 variants one at a time.

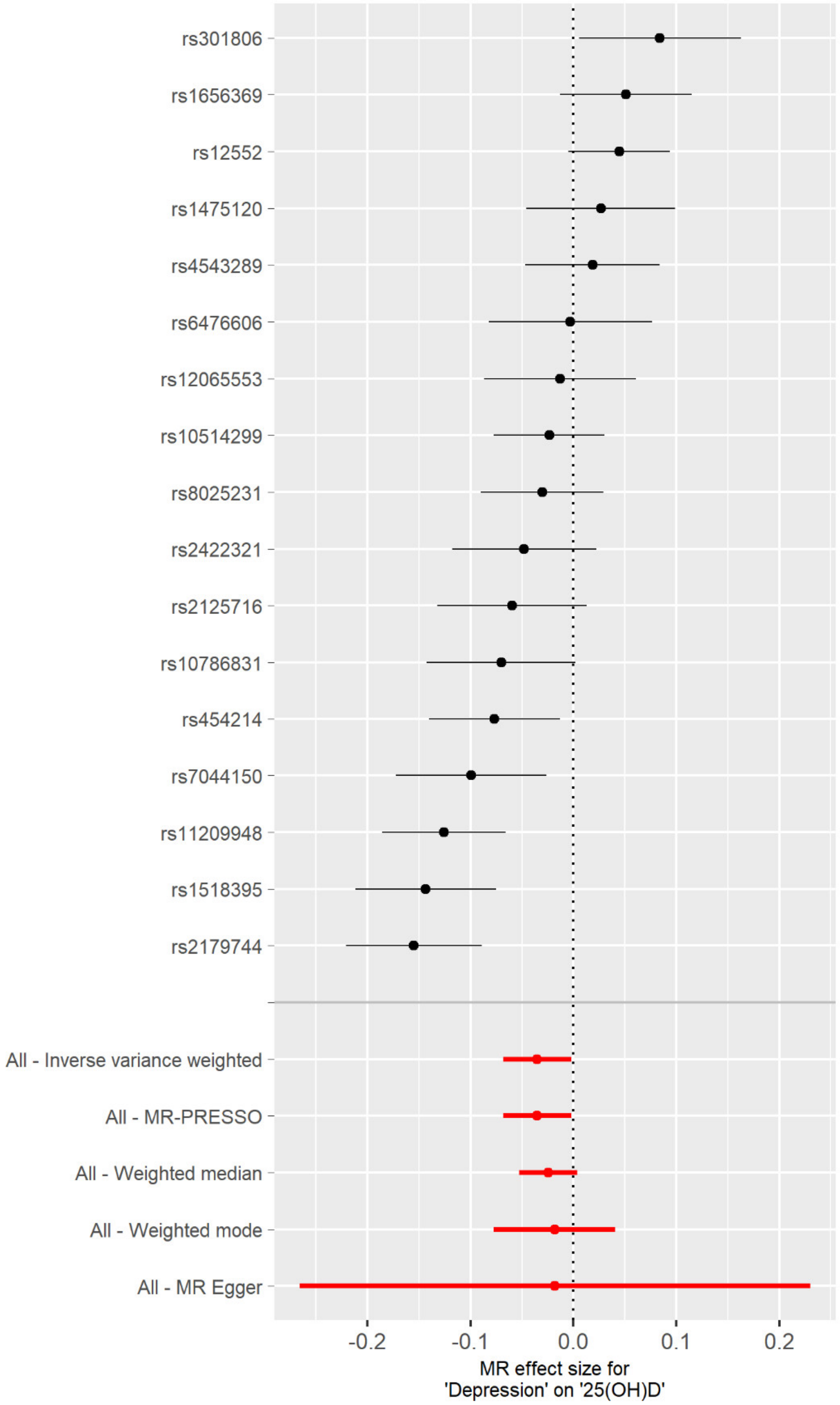

Figure S8. Two-sample MR estimates from different MR approaches using 17 major depression-related genetic variants from Hyde et al (5). MR Egger intercept *p*-value = 0.87.

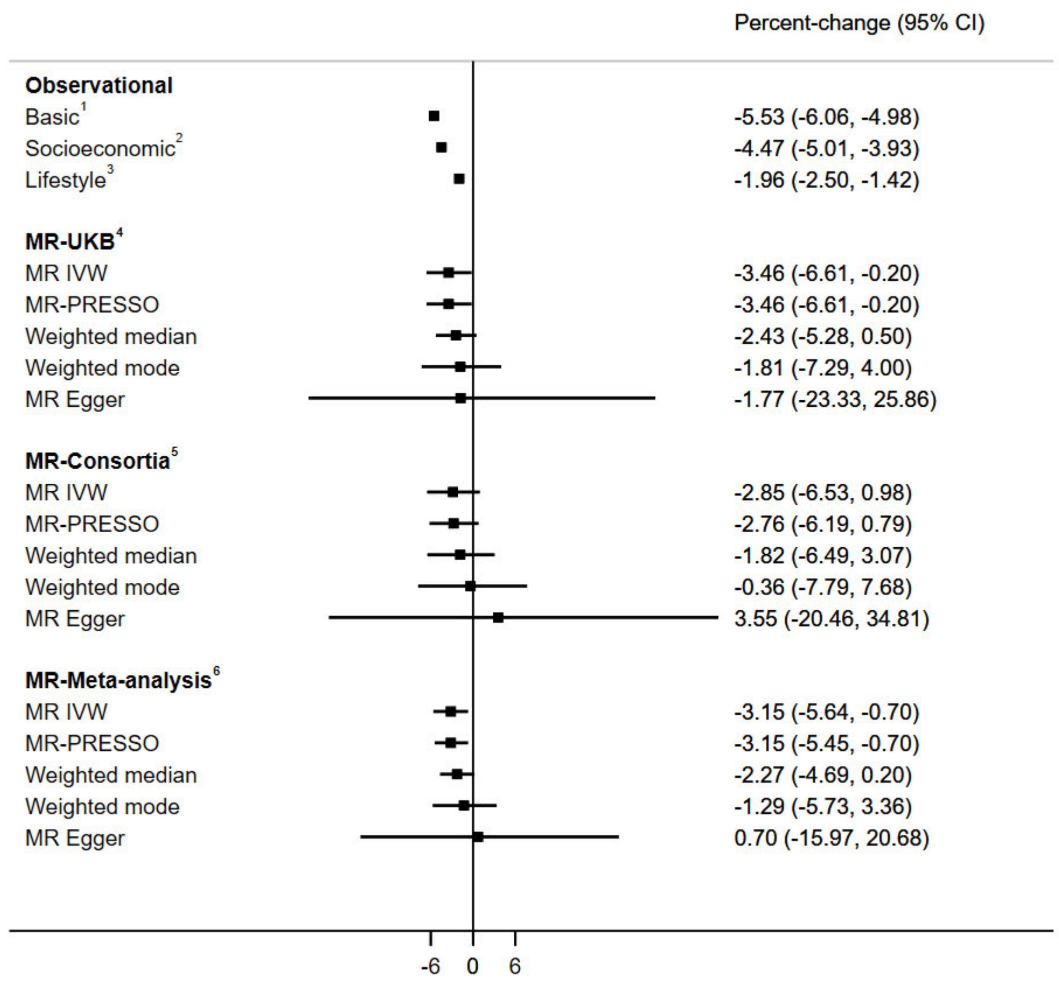

Figure S9. Percent change in serum 25(OH)D associated with depression (Observational), or genetically determined depression (MR) using 17 major depression-related genetic variants from Hyde et al (5). X-axis is percent change. We used 100 × (exp(beta)-1) to get percent increase/decrease

[1] Basic model included adjustment for basic covariates including age, sex, assessment centre, and date of blood sample collected. [2] Socioeconomic model included adjustment for basic and socioeconomic-related covariates including education, Townsend deprivation index, and employment. [3] Lifestyle model included adjustment for basic, socioeconomic and lifestyle-related smoking, alcohol consumption, BMI, physical activity, fish and cheese consumptions, dietary restriction, sun exposure [in summer or winter], use of sun protection, and long standing illness. [4] MR analysis based on variant-serum 25(OH)D association estimates from UK Biobank. [5] MR-analysis based on variant-serum 25(OH)D association estimates from Jiang et al GWAS (3). [6] Meta-analysis of MR estimates from UK Biobank and Jiang et al GWAS. For all MR analysis, variant-depression estimates were from Hyde et al (5). MR-Egger P-intercept (for all), $p < 0.87$.

References

1. Lawlor, D.A.; Harbord, R.M.; Sterne, J.A.; Timpson, N.; Davey Smith, G. Mendelian randomization: Using genes as instruments for making causal inferences in epidemiology. *Statistics in medicine* **2008**, *27*, 1133-1163.
2. Davies, N.M.; Holmes, M.V.; Davey Smith, G. Reading Mendelian randomisation studies: A guide, glossary, and checklist for clinicians. *BMJ* **2018**, *362*, k601.
3. Jiang, X.; O'Reilly, P.F.; Aschard, H.; Hsu, Y.H.; Richards, J.B.; Dupuis, J.; Ingelsson, E.; Karasik, D.; Pilz, S.; Berry, D.; et al. Genome-wide association study in 79,366 European-ancestry individuals informs the genetic architecture of 25-hydroxyvitamin D levels. *Nature communications* **2018**, *9*, 260.
4. Wray, N.R.; Ripke, S.; Mattheisen, M.; Trzaskowski, M.; Byrne, E.M.; Abdellaoui, A.; Adams, M.J.; Agerbo, E.; Air, T.M.; Andlauer, T.M.F.; et al. Genome-wide association analyses identify 44 risk variants and refine the genetic architecture of major depression. *Nat. Genet.* **2018**, *50*, 668-681.
5. Hyde, C.L.; Nagle, M.W.; Tian, C.; Chen, X.; Paciga, S.A.; Wendland, J.R.; Tung, J.Y.; Hinds, D.A.; Perlis, R.H.; Winslow, A.R. Identification of 15 genetic loci associated with risk of major depression in individuals of European descent. *Nat. Genet.* **2016**, *48*, 1031-1036.
6. Revez, J.A.; Lin, T.; Qiao, Z.; Xue, A.; Holtz, Y.; Zhu, Z.; Zeng, J.; Wang, H.; Sidorenko, J.; Kemper, K.E.; et al. Genome-wide association study identifies 143 loci associated with 25 hydroxyvitamin D concentration. *Nature communications* **2020**, *11*, 1647.
